# Focus on the Prevention of Acute Limb Ischemia: Centrality of the General Practitioner from the Point of View of the Internist

**DOI:** 10.3390/jcm12113652

**Published:** 2023-05-24

**Authors:** Iolanda Enea, Eugenio Martelli

**Affiliations:** 1 Emergency Department, S. Anna and S. Sebastiano Hospital, 81100 Caserta, Italy; 2 Division of Vascular Surgery, Department of Cardiovascular Science, S. Anna and S. Sebastiano Hospital, Campania, 81100 Caserta, Italy; eugenio.martelli@uniroma1.it; 3 Department of General and Specialist Surgery Paride Stefanini, Faculty of Pharmacy and Medicine, Sapienza University of Rome, 00161 Rome, Italy; 4 Medicine and Surgery School of Medicine, Saint Camillus International University of Health Science, 00131 Rome, Italy

**Keywords:** acute limb ischemia, peripheral arterial disease, cardiovascular prevention

## Abstract

The thrombotic mechanism, being common to peripheral arterial disease (PAD), acute myocardial infarction (AMI), and stroke, is responsible for the highest number of deaths in the western world. However, while much has been done for the prevention, early diagnosis, therapy of AMI and stroke, the same cannot be said for PAD, which is a negative prognostic indicator for cardiovascular death. Acute limb ischemia (ALI) and chronic limb ischemia (CLI) are the most severe manifestations of PAD. They both are defined by the presence of PAD, rest pain, gangrene, or ulceration and we consider ALI if symptoms last less than 2 weeks and CLI if they last more than 2 weeks. The most frequent causes are certainly atherosclerotic and embolic mechanisms and, to a lesser extent, traumatic or surgical mechanisms. From a pathophysiological point of view, atherosclerotic, thromboembolic, inflammatory mechanisms are implicated. ALI is a medical emergency that puts both limb and the patient’s life at risk. In patients over age 80 undergoing surgery, mortality remains high reaching approximately 40% as well as amputation approximately 11%. The purpose of this paper is to summarize the scientific evidence on the possibilities of primary and secondary prevention of ALI and to raise awareness among doctors involved in the management of ALI, in particular by describing the central role of the general practitioner.

## 1. Introduction

**Definition:** Acute limb ischemia (ALI) is a rapid decrease in blood flow to the lower extremities that requires immediate management to avoid limb loss [1]. It has a poor prognosis for the limb in fact, those with a prior amputation for ALI are known to have a higher frequency of above-the-knee re-amputations than those with no prior amputation for ALI [2], and it has a poor prognosis for life too having a mortality rate of about 15–20% due to concurrent cardiovascular or cerebrovascular disease [3]. ALI has a sudden onset with symptoms lasting less than two weeks and if the onset is greater than 2 weeks it will be referred to as chronic limb ischemia (CLI) both constitute severe forms of peripheral arterial disease (PAD) [4]. The classification according to the severity is an indispensable reference for the therapeutic choice. In this work we will try to summarize the evidence on the importance of prevention, early diagnosis for which the general practitioner and the internist play an essential role.

**Etiology:** The most frequent cause is atherosclerotic and embolic and, to a lesser extent, ruptured aneurysms, dissections, trauma, or surgery itself. In addition to the typically atherosclerotic obstructive form of PAD, small vessel disease (SVD) typical of diabetes and renal failure can also lead to critical/acute ischemia. There are rare forms of ALI that must be considered in the differential diagnosis including conditions of congenital or acquired hypercoagulability (Table 1).

## 2. Epidemiology

Given the heterogeneity of ALI etiology, there are no precise epidemiological data. An estimated 236.62 million (5.56%) people over the age of 25 suffered from PAD worldwide in 2015, and of these, over 73% were in “low- and middle-income countries” (LMICs) [5]. If we consider the prevalence of PAD of about 12% in the American population, the incidence of ALI is 1–2 cases/10,00 people/year [6]. In the “Examining Use of Ticagrelor in Peripheral Artery Disease” study, EUCLID, which enrolled 13,885 patients with PAD, 232 had ALI equal to 1.7% of the total; the patients with ALI were younger, had a low ankle brachial index (ABI), more atrial fibrillation (AF), and prior peripheral revascularization than those with CLI who were 4.6% [7]. Over the years, data from American registries, the most extensive and recent on ALI as far as dated, indicate that the prevalence of ALI has almost halved (from 42.4% to 23%) [8] while one-year mortality and the amputation risk remained unchanged (41% vs. 42%, respectively 14.8% vs. 11%) [9]. Today, ALI is a medical/surgical emergency that in the long run puts both the limb and the patient’s life at risk. Over time it has changed, involving in the past young subjects with mainly rheumatic valvular disease to a current audience of more or less elderly patients with in situ thrombosis or embolism from cardiac arrhythmias mainly AF.

## 3. Complexity of the Disease and Importance of the Multidisciplinary Approach

The complexity of ALI depends on the fact that, in many cases, it is an elderly’s disease. The elderly is characterized by frailty, comorbidities such as diabetes, hypertension, renal insufficiency, which, in addition to prolonged smoking, are among the prevalent risk factors for PAD and ALI. Furthermore, the elderly may not survive a bypass and if they do, they are at increased risk of major cardiovascular events, cardiovascular death, and all-cause mortality [10].

ALI mainly develops on an atherosclerotic process of a native artery. Sometimes thrombosis can affect the end of life. Cancer-associated thrombosis can be another cause of ALI and also in this case mortality is high and the therapeutic strategy will have to be decided with the oncologist.

In young people, in the diagnostic phase, any vasculitis, thrombophilia, paradoxical embolism, etc., should be considered in addition to the presence of atherosclerosis or an embolizing embolic tumor. Whatever the cause, ALI requires rapid medical/surgical intervention, angioplasty, or amputation.

Recently, although endovascular therapy increased from 33% to 83% [11], the recent BEST-CLI trial demonstrated that in patients with autologous saphenous vein availability, surgical treatment is associated with a 32% reduction in events (amputation and/or death) compared to endovascular treatment, while in patients without autologous saphenous vein availability, the outcome of the two surgical and endovascular approaches is superimposable in the long term with a primary outcome of 42.8% in the surgical group and 47.7% in the endovascular group (HR 0.79, 95% CI 0.58–1.06). Moreover, in the same study it is demonstrated that in the group of patients undergoing surgery, octogenarians and patients with renal failure had the same outcome too [12,13].

In 2005, the BASIL trial demonstrated that surgical and endovascular approaches had a similar 3-year amputation-free interval of 57% for surgery and 52% for angioplasty (HR for amputation death 0.89, 95% CI 0.68–1.17) [14].

The ALI, therefore, is to be considered a real failure for the general practitioner and the internist and the role of the vascular surgeon would be greatly reduced if the patient at risk of acute ischemia was taken in charge immediately and was trained in prevention.

The ALI can be reduced either by an improvement of the therapy for atherosclerosis both for a correct AF therapy and for a correct primary prevention.

**Figure 1:**
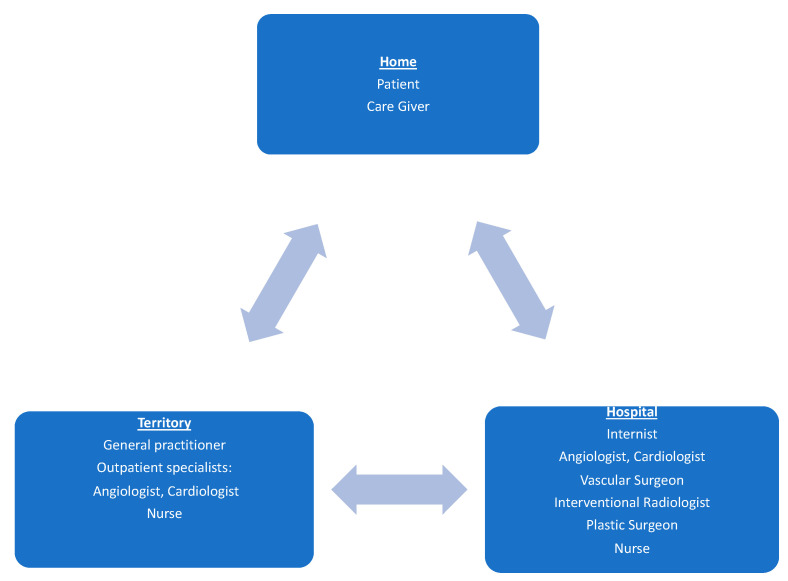
Multi-disciplinary approach to ALI and more generally to PAD. Caption: the multidisciplinary team involves collaboration between care givers, general practitioners, and specialists.

Given the complexity of the pathology, it is understandable that the management of ALI and more generally of PAD must be multidisciplinary, multi-professional (Figure 1). The “Foot care team” must be considered in all phases of the disease and from time to time may include different specialists in relation to the etiology of the ALI but the core will always be made up of a vascular surgeon, radiologist, plastic surgeon, as well as nurses dedicated to the pathology and cannot ignore the involvement of the general practitioner in the area and the hospital internist [15].

## 4. Medical Management

**Figure 2:**
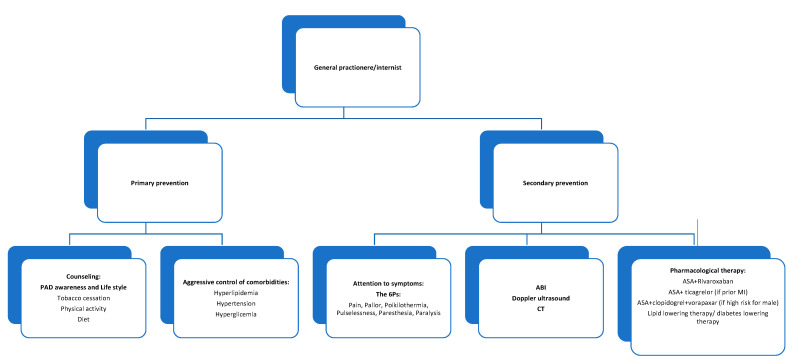
The role of the general practitioner and the internist in PAD/ALI medical management. Caption: actions in which general practitioner and hospital internist may be involved depending on the local organization.

The general practitioner and the internist are called to manage all phases from primary prevention to early diagnosis to secondary prevention of ALI (Figure 2).

### 4.1. The Role in Primary Prevention: PAD Awareness

The first step is to develop awareness of the disease both in the general public and among general practitioners. In fact, it is known that only 26% of patients aged >50 know PAD [16]. Furthermore, only 35% of patients with PAD receive information about the importance of smoking cessation, only 30% of patients take statins [17], only 30% of diabetic patients receive information about the importance of foot care [18].

Already in 2001, the American Heart Association (AHA) launched the first National Action Plan with the aim of improving awareness, diagnosis, and therapy of PAD: increasing awareness improves dialogue between doctors and patients and certainly leads to improved management [19].

Attention should be paid especially to elderly patients: generally, PAD increases with age.

Globally 52.3% of people with PAD are women. Men have been reported to have a higher prevalence of PAD in high-income countries (HICs), whereas women seem to have a higher prevalence of PAD in LMICs. The prevalence of PAD is lower in men than in women until 75 years, at which point it become greater in men than in women [20].

There are not many studies on gender differences in ALI; women seem more likely to have significant femoro-popliteal disease, multi infra inguinal disease than men [21]. It is not known what is the mechanism that leads to a prevalence in females. A study presented the hypothesis of a relationship between metabolic syndrome and inflammation in women since there is a relationship between BMI and C-reactive protein in women while this does not occur in men [22].

### 4.2. The Role in Primary Prevention: Smoking Cessation

The connection between smoking and atherothrombosis is well-known: induction of endothelial damage, reduction of NO, vasodilation, development of an inflammatory and prothrombotic state, induction of insulin resistance and dyslipidemia [23].

The ARIC study demonstrated that smoking is more strongly associated with PAD than other atherosclerotic disease and individuals with >40 pack/years of smoking had four times higher risk for PAD versus two times higher risk for coronary heart disease and stroke [24,25].

There are fewer intervention studies on smoking. One study evaluated the effects of smoking cessation on amputation-free time. In patients who quit smoking, the 5-year amputation-free time was 80% vs. 60% in patients who did not quit (HR 0.43, 95% CI 0.22–0.86) [26].

A further study evaluated the effects of anti-smoking counseling in six sessions with advice on the use of drugs to stop nicotine use in 124 smokers. During the study, the majority of patients took varenicline for smoking cessation. At 6 months, 21.3% of the intervention group remained abstinent from smoking compared with 6.8% of the controls (*p* = 0.023) [27]. The promotion of smoking cessation can therefore take place both with counseling programs and with counseling and the prescription of nicotine replacement therapy such as bupropion or varenicline. Naturally counseling and replacement therapy together are more effective than counseling alone [28,29].

### 4.3. The Role in Primary Prevention: Promote Physical Activity

Until recently it was not known whether physical activity was a determinant of PAD. A recent analysis of the ARIC study showed that higher amount and intensity of physical activity was related to lower risks of hospitalization with PAD and CLI [30]. In patients with PAD, a meta-analysis of 32 randomized trials involving 1835 patients showed that exercise therapy led to a significant improvement in maximum walking distance (mean 82 m; 95% CI 72–92 m). Generally, a training-program included two or three sessions per week of treadmill walking supervised by a trained health professional [31]. Various body resistance training programs improve treadmill walking distance too, but a meta-analysis of five trials showed that treadmill exercise programs improved walking greater than resistance program [32]. About the mechanism, a single study showed that patients with PAD had a fall in plasma nitrite concentrations in response to treadmill exercise, which was not seen in healthy controls [33]. When taken together with the evidence that inorganic nitrate ingestion increases walking distance in patients with intermittent claudication, this finding suggests that recovery of local NO production could contribute to physical therapy benefit [34,35]. So, exercise training is recommended for all patients with symptomatic PAD to improve QOL and to prevent CLI and ALI and the AHA Council on peripheral vascular disease has developed guidelines for optimal exercise programs for patients with symptomatic PAD [36]. The guidelines promote individualized training program for each patient in terms of frequency, intensity, and time; however, they recommend treadmill or other walking-based exercise, involving sessions of 30 to 50 min three times a week for at least 12 weeks. The intensity should be 40–60% of maximum workload at baseline to induce moderate to severe leg pain during steady 5–10 min periods of walking. Then, periodically (every 1–2 weeks), the duration of training session should gradually increase. Lifelong maintenance of at least two sessions of exercise per week is strongly recommended [37]. Most patients cannot access supervised physical therapy but it seems that home training can also be effective provided it is of adequate intensity. In the future it will be necessary to design clinical trials of home-based exercise for PAD building on the home-based exercise interventions already established to meaningfully improve walking outcomes in patients with PAD [38,39].

### 4.4. The Role in Primary Prevention: Weight Control

Patients with body mass index (BMI) between 25 and 29 kg/m^2^ are “overweight”, patients are “obese” if they have a BMI major or equal 30 kg/m^2^. Obesity is to be considered a chronic inflammatory and prothrombotic state. However, an independent association of BMI with atherosclerotic cardiovascular disease may differ by vascular bed. Sex-specific risk factors for atherosclerosis may further modify these associations. The predisposition to atherothrombosis in the case of obesity is multifactorial, it includes inflammation, oxidative stress, dyslipidemia, insulin resistance, endothelial dysfunction, and direct effect on the coagulative cascade [40,41]. The obese have increased leptin which promotes platelet aggregation, it increases C reactive protein, interacts with IL6 contributing to the chronic inflammatory state. Moreover, the elevation of IL1 and TNFα explains the prothrombotic state. In the obese, the increase in PAI1 produced by adipocytes correlates with insulin resistance, hypertriglyceridemia, reduced fibrin clearance, and predisposes to thrombosis [42].

Moreover, adipose tissue is a metabolically active organ, it secretes cytokines, growth factors, it influences energy and vascular homeostasis, and acts on glycolipid metabolism.

The ARIC study showed that BMI is positively associated with incident hospitalized PAD after adjusting for potential confounders, particularly with CLI [43].

Many studies have shown an “obesity paradox” with lower rates of PAD in patients with greater BMI [44]. A recent study on patients with PAD showed elevated odds for PAD in underweight individuals and an association between increasing BMI and PAD in women in contrast to men, who are at increased odds of PAD only with very severe obesity (BMI > 42 kg/m^2^). Obesity and PAD are both more prevalent in women. Obesity itself is part of the metabolic syndrome which includes a cluster of cardiovascular risk factors that are also risk factors for PAD. Therefore, obesity plays a role in the development of PAD and its complications [45]. Therefore, maintaining an optimal body weight and management of obesity-related cardio vascular disease (CVD) risk factors are essential for reducing risk of ALI.

### 4.5. The Role in Primary Prevention: Blood Pressure Control

Current guidelines on diagnosis and management of PAD recommend aggressive management of hypertension. The relation between hypertension and PAD complications is complex. Hypertension is to be considered an indirect risk factor for ALI because the presence of hypertension determines an increase in the average risk of AF of about 30% (RR 1.32, 95% CI 1.08–1.60) [46]. The mechanisms by which hypertension triggers AF are diverse and include activation of the renin angiotensin system, diastolic dysfunction, impaired ventricular arterial coupling, left atrial structural abnormalities; particularly, left atrial enlargement substantially determines electrophysiological changes affecting the atria, a slowing of the impulse conduction velocity together with the shortening of the effective refractory period constitutes the substrate for the triggering of AF [47,48]. AF is predictive of ALI and CLI [7].

The literature on the effect of blood pressure is conflicting: some trials show that antihypertensive treatment in PAD reduces cardiovascular morbidity and mortality or that patients with systolic blood pressure (SBP) less than 120 mmHg have less cardiovascular events [49]. Prior reports described a linear relationship between higher SBP and diastolic blood pressure (DBP) with PAD without an identified risk for lower SBP and DBP [50].

A post hoc analysis of EUCLID trial evaluated the impact of history of hypertension on major adverse cardiac events (MACE) and major adverse limb events (MALE). During 30 months of observation, 13.6% developed MALE and 1.7% ALI. The risk of MALE increased with a combination of SBP 120–129/<70 mmHg and SBP ≥150/≥70 mmHg. The level of 125/80 mmHg was associated with the lowest risk; in the patients with PAD and CAD SBP of 135/145 mmHg was ideal while values below were associated with an increased risk of MACE [51].

Therefore, in established PAD, a clinical history of hypertension is not associated with an increased risk of MALE but low and higher SBP and DBP were associated. This fact emphasizes the importance of blood pressure control rather than the diagnosis of hypertension per se.

### 4.6. The Role in Primary Prevention: Control of Diabetes

Diabetes is an important risk factor of atherosclerotic disease and it increases with the risk of PAD by an OR 1.68 [20]. The risk has been shown to further increase with the duration of the diabetic status [52]. Particularly, diabetes is strongly associated with severe manifestations of PAD [53]. In fact, patients with diabetes have a higher risk of CLI than those without diabetes (HR 5.96, 95% CI 3.15–11.26 and HR 7.45, 95% CI 7.19–7.72) [3]. In addition, those with diabetes are up to five times as likely to require a major lower extremity amputation (LEA), due to higher risk of infection, as well as a higher likelihood of having disease in the distal arteries, thus rendering revascularization technically challenging [54]. Risk factors for developing type 2 diabetes are almost identical to the risk factors associated with atherosclerosis and PAD and include family history, high cholesterol, blood pressure, smoking, inactivity. A population-based Singapore Chinese health study evaluated the interaction of diabetes with hypertension and increased BMI and smoking with the risk of developing CLI and ALI [55]. It showed that diabetes increased the risk of LEA by 13 times (HR 13.61, 95% CI 11.64–15.91) and the risk augmented with the duration of diabetes in those with diabetes for more than 15 years that is a risk of LEA of more than 23 times higher (HR 23.22, 95% CI 18.16–29.67), but hypertension and increased BMI did not further increase LEA among those with diabetes, suggesting a common mechanistic pathway for these risk factors [56]. Diabetic patients can present both macro and micro-angiopathy, isolated or associated: the former is determined by traditional risk factors, such as dyslipidemia and arterial hypertension, whose negative action is increased by hyperglycemia; the second is caused almost exclusively by hyperglycemia. In both, endothelial dysfunction is the first detectable alteration, caused by excessive production of reactive oxygen species (ROS) [57]. The factors explaining the origin of a state of oxidative stress are excessive mitochondrial production of ROS, activation of protein kinase C, activation of the polyol and hexosamine pathways, and increased synthesis of advanced glycation products. Furthermore, coagulation and fibrinolysis processes are also impaired in diabetes [58]. In the EUCLID trial, the sub group of patients with PAD and diabetes are at high risk for cardiovascular and limb ischemic events, even on contemporary therapies. Every 1% increase in HbA1c in patients with a history of diabetes mellitus was associated with a 14.2% increased relative risk for MACE (95% CI: 1.09 to 1.20; *p* < 0.0001) [59]. These data support the conclusion that current strategies inadequately address the increased risk for MACE associated with diabetes, which suggests that more aggressive treatment strategies may be needed along with further research and the development of novel approaches. Meanwhile, the task of family doctors is to educate patients with diabetes and PAD as well as to optimal pharmacological control, also to a careful observation of the extremities, “foot care” [60].

### 4.7. The Role in Primary Prevention: Control of Chronic Kidney Disease (CKD)

CKD is a clinical condition characterized by high morbidity and mortality. It can be diagnosed through the presence of a reduction in estimated glomerular filtration rate (eGFR) < 60 mL/min/1.73 m^2^ and/or an increase in urine albumin excretion, namely albuminuria > 30 mg/g or 30 mg/24 h and/or by the presence of abnormalities in kidney structures persisting for at least 3 months [61]. The eGFR is considered the principal measure of kidney function, whereas albuminuria is a well-assessed marker of kidney damage [62]. CKD patients have an increased risk for cardiovascular (CV) events including PAD [63]. On the global scale, about 50% of CKD patients died of a CV disease [64]. The prevalence of history of CV disease (defined as the positive anamnesis for an episode of MI, stroke, HF, PAD) rose from 30% to 50% in the cohort of MASTERPLAN, Chronic Renal Impairment in Birmingham (CRIB), African Americans Study (AASK), and Italian CKD Multicohort, which enrolled 3.957 CKD patients from multiple diagnoses referred to 128 Italian Renal Clinics, 34% of them had CVD with the following distribution: 15.0% MI, 6.0% stroke, 15.1% PAD, 6.5% HF, and 10% arrhythmias [65]. There is an association between CKD and PAD even after adjustment for the traditional CV risk factors [66]. The risk of PAD is about 6.5 times higher in patients with CKD (eGFR < 60 mL/min/1.73 m^2^) as compared with those without CKD [67]. There is an exponential association between decreasing eGFR/increasing albuminuria and PAD. PAD and CKD probably share common pathophysiological mechanisms: albuminuria is considered a biomarker of endothelial dysfunction. Across the endothelium, albuminuria causes structural and functional alterations such as increase in vascular pressure through direct toxic effect on the tubules, amplifying pro-inflammatory and pro-fibrotic pathways that worse kidney function and contemporary contribute to augment CV risk also via the presence of uremia and volume expansion [68]. Other traditional risk factors play an additional role in determining PAD in CKD patients, among them, smoking habit and arterial hypertension [69], age with a 28% increase every 10 years of age; male gender is a significant predictor of PAD in patient with CKD too, being male conferred a 40% increased probability of developing PAD [70]. The frequency of PAD augment with the severity of KD and about 30% of patients with end stage renal disease (ESRD) have PAD [71]. CKD augment both CLI and ALI and CKD patients are also at high mortality risk [72]. The presence of PAD alone results in a worse prognosis in patients with CKD than the presence of other cardiovascular diseases [73]. But even if these patients are at high mortality risk, they undergo few revascularizations than those without CKD. This situation can be explained considering the complications and worse outcomes after revascularizations. A recent study evaluated the outcomes related to endovascular treatment (EVT) in advanced stages of chronic kidney disease (CKD) and ESRD among hospitalizations with acute limb ischemia (ALI). Advanced CKD stages and ESRD are associated with higher mortality, worse in-hospital outcomes, and higher resource utilization among ALI hospitalizations undergoing EVT (OR = 3.18, 95% CI 2.74–3.69, *p* < 0.0001) compared with group no CKD or stage I/II [74]. CV risk prediction remains suboptimal. Recently, the International Society of Nephrology (I.S.N.) has started a program entitled “closing the gaps” with the aim of encouraging the implementation of novel biomarkers of CV risk in CKD [75]. In the specific context of PAD, improving risk stratification of patients, that is translated in clinical practice into finding CKD patients at high risk of developing PAD, is an urgent need, indeed. The decision to proceed with limb-preservation strategies therefore hinges upon the ability to properly risk-stratify patients. This risk stratification may in fact be different for patients suffering from CKD and chronic limb threatening ischemia (CLTI) [76]. Thus, it is important, for the general practitioner, to consider the importance of following up patients with early-stage CKD to screen them for PAD and taking early preventive measures to slow the turnaround time to the vascular surgeon. A shared decision-making process between the treating physician and patients is desirable when considering endovascular therapy for the treatment of acute limb ischemia in patients with advanced renal disease.

### 4.8. The Role in Primary Prevention: Control of Hyperlipidemia

Lipids are useful for steroid hormone production, bile acid formation, and energy production. They are transported to tissues by lipoproteins that consist of cholesterol, triglycerides (TGs), and phospholipids and protein components named apolipoproteins. There are six major lipoproteins in blood: chylomicrons, very low-density lipoprotein (VLDL), intermediate-density lipoprotein (IDL), low density lipoprotein (LDL); Lipoprotein a (Lp-a), and high-density lipoprotein (HDL). All Apo-B-containing lipoproteins <70 nm in diameter can cross the endothelial barrier, especially in the presence of endothelial dysfunction, where they can become trapped after interaction with extracellular structures [77]. Lipoproteins retained in the arterial wall provoke a complex process that leads to lipid deposition and the initiation of an atheroma. Continuous exposure to Apo-B-containing lipoproteins causes this: other particles are retained in the arterial wall and thus growth and progression of atherosclerotic plaque. Therefore, the risk of experiencing an acute atherosclerotic cardiovascular event rises rapidly as more Apo-B-containing lipoproteins become retained and the atherosclerotic plaque burden increases. The causal role of LDL-C, and other apo-B-containing lipoproteins, in the development of atherosclerotic cardiovascular disease (ASCVD) is demonstrated beyond any doubt by genetic, observational, and interventional studies. Prolonged lower LDL-C is associated with lower risk of ASCVD throughout the range studied, and the results of randomized controlled trials (RCTs) indicate that lowering LDL-C safely reduces CVD risk even at low LDL-C levels [e.g., LDL-C < 1.4 mmol/L (55 mg/dL)]. [78]; the relative reduction in CVD risk is proportional to the absolute size of the change in LDL-C, irrespective of the drug(s) used to achieve such change [79]; no-high-density lipoprotein cholesterol (HDL-C) encompasses all atherogenic (apo-B-containing) lipoproteins, and is calculated as: total cholesterol − HDL-C = non-HDL-C. The relationship between non-HDL-C and CV risk is at least as strong as the relationship with LDL-C. Non-HDL-C levels contain, in essence, the same information as a measurement of apo-B plasma concentrations [80]; HDL-C is inversely associated with CVD risk. Very high HDL-C levels may signal an increased CVD risk. HDL-C is nonetheless a useful biomarker to refine risk estimation using the SCORE2 and SCORE2 OP algorithms [81]. The SCORE2 algorithm cannot be used for patients with a genetic lipid disorder, such as familial hypercholesterolemia (FH). Specific LDL-C thresholds and targets are recommended irrespective of estimated CV risk for patients with FH or other lipid disorders. Dyslipidemia, particularly hypercholesterolemia, is associated with an increased prevalence of PAD. Elevated HDL values have been shown to be protective for PAD. Hypertriglyceridemia and elevated Lp(a) values are also risk factors for PAD. High-lipoprotein(a) (Lp-a) levels are involved in the development of cardiovascular events, particularly in AMI, stroke, and PAD and they are independently associated with an increased risk of a major adverse limb event in hospitalized patients [82]. So, the Lp(a) measurement needs to be taken into account to improve lower-limb vascular risk assessment. This provides the rationale for encouraging a healthy lifestyle to maintain low levels of Apo-A, Apo-B-containing lipoproteins throughout life to slow the progression of atherosclerosis; it also explains the motivation to recommend treatment to lower LDL-C and other Apo-B-containing lipoproteins, for both the primary prevention of ASCVD and the secondary prevention of recurrent CV events [83].

### 4.9. The Role in Primary Prevention: Rhythm Control

It is well-known that a low ABI, a previews revascularization, and AF are predictors of ALI in patients with PAD [7] The prevalence of AF in patients with PAD is 10–13% [4]. It has been reported that patients with AF and PAD have a higher incidence of adverse events; among them, the presence of PAD is significantly associated with a 1.3–2.5-fold increased risk of stroke, and the risk of thrombotic events, including ischemic stroke, is increased up to two-fold [46]. The current guidelines recommend oral anticoagulant (OAC) therapy instead of antiplatelet therapy (APT) for patients with AF and PAD; meanwhile, the combination of OAC therapy and APT can be considered for patients with AF and PAD undergoing intravascular revascularization [84]. However, OAC therapy combined with APT may increase severe bleeding, including intracranial bleeding [85].

## 5. The Role of the General Practitioner in PAD Risk Stratification and Early Diagnosis of ALI

### 5.1. Attention to Symptoms and Signs

The general practitioner, in addition to paying attention to the family anamnesis, personnel anamnesis with regard to the patient’s physical activity and diet, the presence of comorbidities (hypertension, diabetes, dyslipidemia, heart failure, renal insufficiency, AF) with particular attention to the onset of symptoms such as intermittent claudication, must therefore act aggressively against any risk factors for PAD, trying to identify early symptoms and signs of the progression of the disease. Symptoms of acute arterial occlusion appear in the affected limb (usually your leg). Healthcare providers refer to the symptoms as the “six Ps” (Table 2).

In clinical practice, it is rare for all six signs to occur together unless severe ALI occurs in a patient with normal arteries perhaps due to an embolic episode.

The objective examination of both legs must meticulously detect chromatic variations of the skin, any skin lesions, temperature difference in the limbs, palpation of the arterial pulses (carotid, femoral, popliteal, dorsal pedis, posterior and anterior tibial), listening for any abdominal murmurs; evaluation of possible diabetic neuropathy. Loss of sensory and motor function are symptoms of a limb in need of immediate revascularization. Patients with deep vein thrombosis or neurological impairment may have similar symptoms of ALI. Naturally, considering the possible cardiac origin of the emboli, a cardiac examination is necessary. Diagnosing ALI is usually straightforward given the symptoms. In any case it is necessary to be very rapid in the diagnosis in order to be able to direct the treatment and avoid losing the limb. The classification according to the severity is an indispensable reference for the therapeutic choice. Rutherford classification is used to evaluate whether the ischemic limb is threatened, viable, or irreversible ischemic and thus lead to the management of the leg [86] (Table 3).

### 5.2. The Use of the Ankle Brachial Index (ABI) in Risk Stratification

ABI identify is a marker of cardiovascular risk (a value less or equal 90 is associated with a two-fold increase in CV death) and is a simple, useful, inexpensive tool for the diagnosis and surveillance of lower extremity arterial disease (LEAD); since it is known that an ABI of less than 60 is associated with a greater risk of hospitalization for ALI, it is useful in the risk stratification too. This simple method should be used by every general practitioner in the following conditions: (a) all men and women over the age of 65; (b) men and women under the age of 65 at high cardiovascular risk; (c) patients over 50 years of age with a family history of LEAD; (d) patients with known PAD and CAD; (e) patients with CKD, HF, AAA; (f) patients with reduction or abolition of arterial pulses; (g) symptomatic patients [1]. To measure the ABI, the patient is placed in the supine position with the cuff positioned just above the ankle. After 5 to 10 min, blood pressure is measured with a doppler probe (5 to 10 MHz) at the posterior and anterior tibial arteries of each foot and at the brachial artery of each arm. To avoid pressure oversights, manual pressure gauges are to be preferred. The ABI of each leg is calculated by dividing the highest ankle SBP by the highest arm SBP. Recently the VIVA trial showed the benefit of using ABI, blood pressure control, and ultrasonography for the screening of vascular disease and to optimize vascular preventive therapy with a reduction of 7% in mortality [87]. Unfortunately, the ABI is yet under-used.

## 6. The Importance of Optimizing Medical Therapy before and after Revascularization

### 6.1. Medical Therapy and Vascular Protection

**Figure 3:**
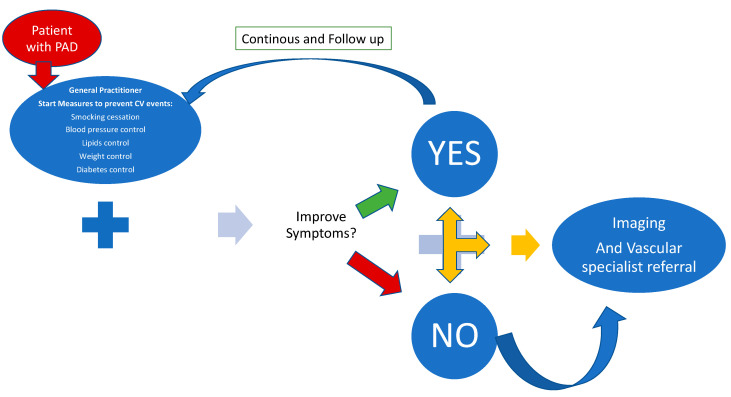
The journey of the patient with PAD to prevent ALI. Caption: The patient with PAD turns to the treating physician who implements secondary prevention measures including physical activity supervision and, if these have no effect, the patient turns to diagnostic imaging and to the vascular specialist.

The patient with PAD must be involved in the management of the disease and must collaborate with the family doctor (Figure 3).

After the diagnosis of ALI, the patient must be referred by the general practitioner to the vascular surgeon immediately, because any use of diagnostic imaging would be a waste of time which delays any therapeutic decision and could lead to the loss of the limb. The goals of ALI care include urgent surgical or endovascular revascularization [88] to prevent disabling amputation or to limit the level of amputation, with endovascular procedures increasingly utilized [89]. However, CLI is frequently accompanied by several other comorbidities, thus, medical therapy and risk factor modification are crucial for disease management and prevention of major cardiovascular events. Sub-optimal medical therapy for comorbid condition have been associated with up to 26% all-cause mortality rates within the first year of CLI diagnosis [90]. The role of the general practitioner and of the internist will be fundamental both for preventive purposes and after the revascularization to avoid further complications and prevent recurrences.

**Figure 4:**
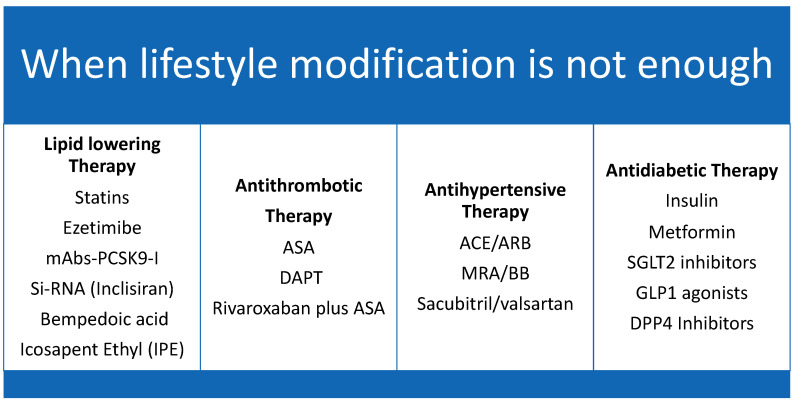
The 4 Pillars of vascular protection therapy. Caption: Medical therapy for vascular protection.

Therefore, the pillars in preventing limb loss in the PAD patient are: aggressive management of modifiable risk factors, timely referral to the revascularization specialist, and proper use of medical therapy in both prevention and follow-up post-revascularization [91], Figure 4.

Primary and secondary prevention of PAD includes antithrombotic therapy, lipid-lowering therapy, blood pressure, and diabetes control [92].

Studies on PAD are outnumbered by those on CAD and stroke, and many times data on PAD therapy have been extrapolated from studies of other vascular systems; however, in the last period, the literature has been enriched with interesting studies that have specifically studied the impact of medical therapy on PAD: EUCLID, FOURIER, COMPASS [93,94,95]

A sub-analysis of the EUCLID study showed that patients with CLI, the most severe form of PAD with high mortality and morbidity, are under-treated, particularly with regard to statin therapy [7].

Yet it is now clear that lipid-lowering therapy does not present a J curve even in the peripheral vascular district, supporting the thesis according to which dyslipidemia is not a simple cardiovascular risk factor but a causal agent of atherosclerosis and as such should be treated by supporting the concept “the lower, the earlier, the better”.

The GGLL 2017 already recommended the use of statins in all patients with symptomatic PAD (1A) with the aim of reducing LDL-C to a level below 70 mg/dL or to reduce the baseline value by 50% (1C) [1]. However, after the GGLL ESC 2019 on cardiovascular prevention, the paradigm of statin-based therapy has changed, they recommend that the correct choice of therapy must derive from the baseline value of the patient’s cholesterol, from the individual risk stratification from the target to be achieved based on risk [92,96] (Table 4).

The guidelines identify the patient with multi-district vascular disease that is symptomatic or evident on diagnostic imaging, at very high risk with a target LDL-C of 55 mg/dL with a cholesterol reduction of at least 50%, if the patient has already had a peripheral vascular event as well as central and recurrent events, at extreme risk indicating as goals, 40 mg/dL in each case with a reduction of at least 50% compared to baseline values. Another novelty for PAD concerns the use of the so-called vascular dose of rivaroxaban, a direct anticoagulant, associated with ASA, which reduced the composite end point of stroke, heart attack, and cardiovascular death by 28%, and MALES by 46%, amputations by 70% [97].

### 6.2. Lipid Lowering Therapy

There are several lipid-lowering drugs: statins, fibrates, monoclonal antibodies PCSK9 inhibitors (mAbs-PCSK9-I), inclisiran, bempedoic acid, eicosapentaenoic acid. Simvastatin reduces the risk of MACE compared with placebo in patients with PAD (HR 0.78, 95% CI 0.71–0.85) [98]. Statins are first line therapy in PAD (1A) according to the last ESC guidelines on PAD; they reduce LDL-C, slow the progression of atherosclerosis, and reduce morbidity and mortality associated with coronary, cerebrovascular, and peripheral vascular events [1]. They act by inhibiting 3-beta-hydroxy-methyl-glutaryl coenzyme, a reductase responsible for the synthesis of mevalonic acid, blocking cholesterol production [99]. They have different potency in reducing LDL cholesterol, so we distinguish low-, moderate-, high-intensity statins (Table 5).

Patients with PAD are at high or very high cardiovascular risk, therefore depending on how far the baseline cholesterol value deviates from the recommended target, it will be necessary to use high-intensity statins as monotherapy immediately or in combination.

**Among lipid-lowering therapy other than statin therapy,** a study evaluated the effect of bezafibrate on cardiovascular events in patients with PAD and demonstrated a significant reduction in triglycerides level by 23.3% and in LDL-C level by 8.1%, as well as a rise in HDL-C level by 8%. However, the clinical benefits were not as satisfactory: bezafibrate treatment showed a reduction in the incidence of non-fatal coronary events, but failed to prove any benefits regarding coronary heart disease and stroke [100].

The Fenofibrate Intervention and Event Lowering in Diabetes (FIELD) study showed that the use of fenofibrate in patients with diabetes did not reduce mortality but it reduced total cardiovascular events [101] and it was associated with a lower risk of amputations, particularly minor amputations without known large vessel disease (HR 0.64, 95% CI 0.44–0.94) [102]. The Action to Control Cardiovascular Risk in Diabetes (ACCORD)-lipid trial was designed to investigate the combination of fenofibrate plus a statin compared with statin monotherapy in patients with diabetes mellitus, it showed no significant difference between the two groups to reduce the risk of cardiovascular disease [103]. However, the combination therapy was associated with a 31% lower event rate in a subgroup (17% of the total) with significant hypertriglyceridemia and low HDL-C [104]. Post hoc analyses of fibrate trials have suggested that patients with high triglycerides levels and low HDL-C levels benefited from fibrate therapy even when the overall trials results were neutral. [105]. Recently, the Pemafibrate to Reduce Cardiovascular Outcomes by Reducing Triglycerides in Patients with Diabetes (PROMINENT) trial precisely picked this patient population and still did not show a benefit; it showed that, as compared with placebo, pemafibrate lowered triglyceride levels by 26.2% and increased HDL cholesterol levels by 5.1%, it did not reduce the incidence of events that made up the primary efficacy end point (a composite of nonfatal myocardial infarction, ischemic stroke, coronary revascularization, or death from cardiovascular causes) [106]. Probably, the lack of efficacy despite triglyceride lowering may be largely due to a lack of an overall decrease in the apolipoprotein B level. It is likely that the apolipoprotein B-lowering effects of fibrates are negated in the presence of moderate-to-high-intensity statins, as seen in this trial. Probably, lowering triglycerides without decreasing apolipoprotein B level is not sufficient to produce significant decreases in the risk of atherosclerotic cardiovascular disease and fibrates should not be used to reduce the risk of atherosclerotic cardiovascular disease among statin-treated patients [107].

A marked reduction in apolipoprotein B levels would therefore be an important early surrogate to follow in such cases. Even the studies on **eicosapentaenoic acid** would lead in this direction. In the REDUCE-IT (Cardiovascular Risk Reduction with Icosapent Ethyl-Intervention for hypertriglyceridemia) trial of high ischemic risk patients with elevated triglyceride levels despite statin use, icosapent ethyl induced a significantly lower incidence of primary endpoint events compared to the placebo group with a relative cardiovascular risk reduction of 25% and an absolute risk reduction of 48%. This reduction in the cardiovascular risk was unrelated to changes in triglyceride levels, but likely with apolipoprotein B levels 9.7 percentage points lower in the icosapent ethyl group compared to the placebo group [108]. In contrast, the effect of high dose ω-3 fatty acids vs corn oil on major adverse cardiovascular risk: the STRENGTH randomized clinical trial and a secondary analysis of the same study on the association of the achieved ω-3 fatty acids and major cardiovascular events did not show a significant decrease in apolipoprotein B levels and/or a decrease in the incidence of cardiovascular events among patients who received Epanova, an eicosapentaenoic acid–docosahexaenoic acid combination [109,110]. There are some differences between the two studies both in the study population and in the drugs used. In the REDUCE-IT trial most of the population, about 70% was in secondary prevention; in the REDUCE-IT study, a purified formulation of high-dose eicosapentaenoic acid was used while the STRENGTH trial used a combination of EPOA and DHA [111]. Taken together, these findings highlight the importance of net lowering atherogenic lipoprotein levels rather than lowering triglyceride levels per se. However, more research is needed to explain the role of apolipoprotein B and the results of the two studies.

Colestipol plus niacin in the Cholesterol Lowering Atherosclerosis Study (CLAS) also led to a decrease in serum triglycerides and an increase in high-density lipoprotein cholesterol (HDL-C), along with a decrease in LDL-C, which correlated with a slower progression of atherosclerosis in femoral arteries, although less marked than expected, considering the previous results in coronary artery disease [112].

**Ezetimibe**, another lipid-lowering drug which proved to lower LDL-C when added to statins, was also assessed in patients with PAD [113]. One study tried to evaluate the evolution of atherosclerotic plaques in the superficial femoral artery. Interestingly, adding ezetimibe to patients previously treated only with statin led to a progression in peripheral atherosclerosis, despite a 22% decrease in LDL-C. These results correlate with those of a different study which compared the effects of niacin added to statin to ezetimibe added to statin therapy. Although the combination of ezetimibe plus statin led to a greater reduction in LDL-C than the use of niacin in addition to statin, there was a paradoxical increase in the carotid intima-media thickness in patients with lower LDL-C levels among those treated with ezetimibe. Although this study did not assess peripheral arteries, it seems that ezetimibe does not reduce the cardiovascular risk and does not prevent the progress of disease in patients with PAD [114].

**mAbs-PCSK9-Inhibitors.** The FOURIER trial showed that therapy with evolocumab a PCSK9 reduced the risk of MACE in PAD patients (HR 0.73, 95% CI 0.59–0.91), it reduced the risk of MALE (HR 0.58, 95% CI 0.38–0.88), so the reduction of LDL seems to reduce the progression of atherothrombosis [73]. Alirocumab in patients with poli-vascular disease did not reduce the rate of the primary outcome but it reduced the risk of all causes of death by 16.2% (95% CI 5.5–26.8%) [115].

**Bempedoic Acid** is a prodrug: it requires conversion by the very long chain acyl CoA synthetase 1 into a CoA-thioester, the active metabolite. The CoA-thioester inhibits adenosine triphosphate-citrate lyase (ACL) that is the cholesterol biosynthetic pathway upstream of HMG-CoA reductase, in this way it inhibits the mevalonate pathway, depletes cellular cholesterol, and up regulates hepatic LDL receptors to lower circulating LDL cholesterol levels. By acting only in the tissue with ACL that is not contained in muscular tissue, bempedoic acid can offer the advantage over statins to avoid muscular symptoms or iperglycemia even if there are some collateral effects also with bempedoic acid such as tendon rupture, increased uric acid, gout, and reduced glomerular filtration rate which are not seen in the statin use. The preferential use of bempedoic acid is in patients with LDL-C levels >70 or 100 mg/dL despite maximum tolerated statins and in those with statin intolerance. Data from CLEAR outcomes trial shows that Bempedoic monotherapy lowers the LDL-C level up to 28% and 16% in patients receiving the maximum tolerated doses of statins. It also determines a reduction in the level of C reactive protein [116,117].

Data from the CLEAR Outcomes Study show that bempedoic acid lowers MACE by reducing LDL-C levels [116]. In detail, in 13,970 patients at high risk of atherosclerotic vascular disease, intolerant to statins, assigned to receive bempedoic acid 180 mg daily or placebo, a 21% reduction in LDL-C levels was observed corresponding to 13% less risk of MACE defined as a four-component composite of cardiovascular death, non-fatal MI, non-fatal stroke, and coronary revascularization over a median of 3.4 years [117]. Bempedoic acid reduced the risk of secondary endpoint events, including cardiovascular death, non-fatal MI, non-fatal stroke, fatal MI, and coronary revascularization. Interestingly, a numerically greater effect of bempedoic acid on the primary endpoint was observed for 30% of patients in the primary prevention cohort compared with 70% of patients in the secondary prevention cohort. Moreover, similar effects were seen when adding bempedoic acid to ezetimibe and a very low dose of a statin. Combining IPE with ezetimibe results in a 35% to 40% reduction in LDL cholesterol levels. Thus, bempedoic acid is now on the list of evidence-based alternatives to statins in patients with known atherosclerotic disease or at high risk for vascular disease or who are unable or unwilling to take statins [118].

**Inclisiran** is a small interfering ribonucleic acid (siRNA) that prevents hepatic PCSK9 production [119]. Inclisiran with maximally tolerated statins has been approved to reduce LDL-C in patients with primary hypercholesterolemia or mixed dyslipidemia by the European Medicines Agency in patients with familial hypercholesterolemia or clinical ASCVD. The ORION phase III trials showed that the use of inclisiran reduce LDL-C of 1.37 mmol/L (50.6%) and composite MACE (OR 95% CI 0.74, 0.58–0.94) but not fatal and non-fatal MI and stroke (OR 95% CI 0.80, 0.50–1.27 and 0.86, 0.41–1.81 respectively) [120,121]. A 4-year open label extension of the ORION 1 trial demonstrated that inclisiran reduced LDL C and PCSK9 concentrations and is well-tolerated [122]. There are no specific studies on MALE and there are only preliminary data on MACE from a pre-specified pooled patient-level analysis of high-risk patients with elevated LDL-C from the ORION studies 9, 10, 11 demonstrating that adding inclisiran to lipid-lowering therapy significantly reduced MACE composite but not limited to fatal and non-fatal MI or fatal and non-fatal stroke, suggesting that this approach has potential cardiovascular benefits [121], but the ORION outcome studies (ORION-4 NCT03705234 and VICTORIAN-2P NCT0503428) are ongoing, therefore we do not have yet definitive data about MACE reduction. Therefore, in patients with multivessel disease, inclisiran may be used in adjunction with statin and ezetimibe.

### 6.3. Antithrombotics

Antiplatelet therapy is controversial in asymptomatic patients, while it is recommended on a grade of evidence 1C in patients with symptomatic PAD with preference for clopidogrel vs ASA.

A post hoc analysis of the CAPRIE study showed that 75 mg clopidogrel has to be preferred over 100 mg ASA because it reduces the risk of MACE (RR 0.76, 95% CI 0.64–0.91) [123]. There are no differences between 75 mg clopidogrel and 90 mg twice a day ticagrelor [93].

The COMPASS trial showed that low dose rivaroxaban, 2.5 mg twice a day plus 100 mg ASA reduced the risk of MACE better than aspirin alone in 7.470 patients with PAD (HR 0.72, 95% CI 0.57–0.90), major adverse limb event (HR 0.54, 95% CI 0.35–0.84), and major amputation (HR 0.30, 95% CI 0.11–0.80) [124]. The dual therapy with low dose rivaroxaban and aspirin reduces the risk of major cardiovascular events also in patients with symptomatic PAD undergoing revascularization vs aspirin alone (HR 0.85, 95% CI 0.76–0.96) [125]. These results come at the cost of an increase in bleeding. In conclusion there is strong evidence for the use of dual therapy in patients with symptomatic PAD excluding those with high risk of bleeding.

### 6.4. Antihypertensives

From the International VErapamil SR/Trandolapril (INVEST) study [50] we know that caution should be exercised to avoid an SBP decrease below 110–120 mmHg since a J shape relationship between SBP and cardiovascular events has been reported in LEAD patients. A salt intake of 5–6 g/day is advisable. Diuretics, betablockers, calcium antagonists, angiotensin converting enzyme inhibitors (ACE), and angiotensin receptor blockers (ARB) can be used alone or in combination therapy. The ONTARGET study [126] showed that ACE and ARB significantly reduced CV events in patients with PAD so they are to be preferred in these patients above all in patients with CLI for secondary prevention [126,127]. In fact, in patients with CLI, the use of ACE and ARB is associated with a reduction of MACE and mortality without any effect on limb outcome [128]. If patients with LEAD and hypertension have HF they can use beta blockers, in particular nebivolol, because they are safe and without any negative effect on WD [129]. A RCT including 128 patients with IC and hypertension studied metoprolol and nebivolol. Both drugs were well-tolerated and improved WD especially nebivolol (improvement in pain free WD +34% (*p* < 0.003) vs. +17% for metoprolol *p* < 0.12) [130].

In hypertensive patients it is recommended to maintain blood pressure below 140/90 mmHg, possibly with ACE and ARB with grade 1A evidence (IIaB).

### 6.5. Anti-Diabetes Drugs

Effectively controlling diabetes helps to prevent its deleterious effects on the cardiovascular system. In general, “intensive” blood glucose control leads to a lower risk of amputation than normal blood glucose control [131]. It has recently been shown that in patients with diabetes and PAD, the SGLT2 inhibitor empaglifozin reduces the risk of cardiovascular death (HR 0.57, 95% CI 0.37–0.88) and mortality from all causes (HR 0.62 95% CI 0.44–0.88) [132]. The VERTIS trial showed that ertiglifozin induced similar risk of MACE [133]. In another trial DECLARE TIMI 58 dapaglifozin did not reduce MACE but reduced the risk of cardiovascular death or hospital admission for heart failure. There were no differences in the risk of amputation and limb ischemic event in the dapaglifozin group and the placebo one [134]. Canaglifozin appears to reduce the risk of MACE (HR 0.86 95% CI 0.75–0.97) and to increase the risk of amputation (HR 1.97 95% CI 1.41–2.75) [135] but these data have not been confirmed by successive studies. In fact, a post hoc analysis of data from the CANVAS program (N = 10.142) and the CREDENCE trial (N = 4.401) showed that canagliflozin does not increase the risk of MALE regardless of baseline PAD history and that the absolute benefits of canagliflozin are greatest in patients with PAD [136].

### 6.6. Cilostazol

It is a promising phosphodiesterase 3 inhibitor but the overall intake is variable probably owing to mixed results on efficacy, tolerance, adverse effects. In particular, cilostazol increases CAMP levels and upregulates NO production [137,138]. A meta-analysis of data showed that cilostazol reduces the risk of amputation (HR 0.42 95% CI 0.27–0.66) and repeat revascularization (RR 0.44, 95% CI 0.37–0.52) in patients who underwent revascularization [139]. But another meta-analysis showed that cilostazol did not improve WD in patients with IC [140]. It is contraindicated in HF; and it has many collateral effects: palpitations, dizziness, bleeding [141].

### 6.7. ALI Prevention, PAD Management, and Other Arterial Basins

The general practitioner, as well as other local specialists, has to be aware that lesions present at a peripheral level can generally be present in other arterial districts, above all in the brain and heart [142]. These lesions can occur asymptomatically and can lead to the death of the patient due to MI or stroke [143]. Similarly, in patients with AMI and stroke, the evaluation of the residual cardiovascular risk cannot disregard the evaluation of the peripheral arterial status [144]. The approach we suggest to adopt is, therefore, a holistic approach to the patient with atherosclerotic disease that takes into account the global cardiovascular risk at every moment of the patient’s life.

## 7. Conclusions

Primary and secondary prevention of PAD is the useful tool to reduce the incidence of ALI. In the context of multi-specialist teams, the general practitioner is certainly the most suitable for promoting both primary and secondary prevention in collaboration with specialists. They include the control of the main risk factors: smoking, hypertension, dyslipidemia, renal insufficiency; the identification of subjects at risk of evolution toward CLI and ALI; the use of ABI; the promotion of correct lifestyles and drugs in secondary prevention always involving the patient and care giver. Properly designed registry and prospective studies could be the next step to evaluate the impact of primary and secondary prevention on the outcome of patients with PAD at risk of ALI.

## Figures and Tables

**Table 1:** Unusual causes of ALI and corresponding mechanisms.

CAUSE	MECHANISM
Antiphospholipid Syndrome	Acquired thrombophilia
Hyperhomocysteinemia	Acquired thrombophilia
Heparin induced Thrombocytopenia	Immune
Disseminated intravascular coagulation	Hypercoagulability
COVID 19	Hypercoagulability
Malignancy	Hypercoagulable state and embolic material
Vasculitis	Inflammation
Low cardiac output	Low blood flow in the extremities during hypotension shock sepsis
Paradoxical embolism	Atrial septal defect
Advential cystic disease	Cyst occluding blood flow in the artery
Acute compartment syndrome	Tissue compressing artery

**Table 2:** 6P symptoms.

1.	PAIN
2.	PALLOR
3.	POIKILOTHERMIA
4.	PULSELESSNESS
5.	PARESTHESIA
6.	PARALYSIS

**Table 3:** ALI severity Rutherford classification.

Grade	Category	Doppler	
		Arterial	Venous
I	Limb viable	Audible	Audible
IIA	Limb marginally threatened, salvage- able if promptly treated	Often inaudible	Audible
IIB	Limb immediately threatened, salvageable with revascularization	Inaudible	Audible
III	Limb irreversible damaged	Inaudible	Inaudible

**Table 4:** Cholesterol target based on the level of cardiovascular risk. Caption: Patients with PAD are by definition very high risk.

Cardiovascular Risk Level	LDL Cholesterol Target
Recurrent ischemic events	<40 mg/dL
Very high risk or secondary prevention	<55 mg/dL and at least a 50% reduction in baseline LDL-C levels
High risk	<70 mg/dL and at least a 50% reduction in baseline LDL-C
Moderate risk	<100 mg/dL
Low risk	<116 mg/dL

**Table 5:** Type and dosage of moderate- and high-intensity statins. Caption: High-intensity and moderate-intensity statins induce 50% and 30% of LDL cholesterol levels, respectively.

Moderately-Intensive Statins: 30% LDL-C Reduction	High-Intensive Statins: 50% LDL-C Reduction
Atorvastatin 10–20 mg	Atorvastatin 40–80 mg
Rosuvastatin 5–10 mg	Rosuvastatin 20–40 mg
Simvastatin 20–40 mg	
Pravastatin 40–80 mg	
Lovastatin 40 mg	
Fluvastatin 80 mg	
Pitavastatin 2–4 mg	

Several studies including PAD subjects demonstrated that further reduction in LDL-C based on the high intensity statin therapy could significantly reduce cardiovascular and cerebrovascular events [79].

## Data Availability

Not applicable.
